# Microbiota and Glucidic Metabolism: A Link with Multiple Aspects and Perspectives

**DOI:** 10.3390/ijms241210409

**Published:** 2023-06-20

**Authors:** Tiziana Ciarambino, Pietro Crispino, Gaetano Leto, Giovanni Minervini, Ombretta Para, Mauro Giordano

**Affiliations:** 1 Internal Medicine Department, Hospital of Marcianise, ASL Caserta, 81037 Caserta, Italy; 2 Internal Medicine Department, Hospital of Latina, ASL Latina, 04100 Latina, Italy; 3 Department of Experimental Medicine, University La Sapienza Roma, 00185 Rome, Italy; 4 Internal Medicine Department, Hospital of Lagonegro, AOR San Carlo, 85042 Lagonegro, Italy; 5 Internal Emergency Department, Hospital of Careggi, University of Florence, 50121 Florence, Italy; 6 Department of Medical Science, University of Campania, L. Vanvitelli, 81100 Naples, Italy; mauro.giordano@unicampania.it

**Keywords:** diabetes, insulin resistance, microbiota differences

## Abstract

The global prevalence of overweight and obesity has dramatically increased in the last few decades, with a significant socioeconomic burden. In this narrative review, we include clinical studies aiming to provide the necessary knowledge on the role of the gut microbiota in the development of diabetic pathology and glucose-metabolism-related disorders. In particular, the role of a certain microbial composition of the fermentative type seems to emerge without a specific link to the development in certain subjects of obesity and the chronic inflammation of the adipose tissues, which underlies the pathological development of all the diseases related to glucose metabolism and metabolic syndrome. The gut microbiota plays an important role in glucose tolerance. Conclusion. New knowledge and new information is presented on the development of individualized therapies for patients affected by all the conditions related to reduced glucose tolerance and insulin resistance.

## 1. Introduction

**Figure 1:**
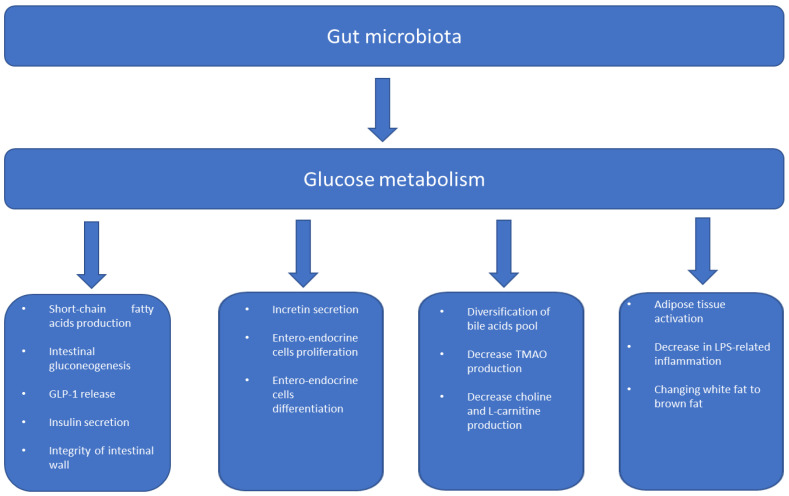
Gut microbiome and metabolism.

From an epidemiological point of view, the pathological conditions related to overweight and obesity are constantly increasing in all geographical areas, with socio-economic repercussions terms of mortality related to diseases such as dyslipidemia, hypertension, and type 2 diabetes mellitus (T2D) [1,2]. In particular, so-called acquired diabetes mellitus or T2D originates in the persistence of high daily glucose levels compared to normal values, due to the resistance of the target tissues to the effect of insulin [3,4]. The pathogenesis of polyfactorial diabetes depends on the mechanisms that determine peripheral insulin resistance, as well as on the distribution of body fat, particularly central body fat, which is correlated more strongly with the metabolic syndrome (MetS) as it actively enters glucose homeostasis, the progressive dysfunction of the pancreatic beta cells [5,6]. Furthermore, there are connections between the metabolic alterations between pancreatic function and hepatic function that are not fully understood, as well as the mechanisms that determine insulin resistance in other peripheral tissues, including skeletal muscle and adipose tissue. The complexity of this relationship between the various organs united by their sensitivity to insulin should favor correct glucose metabolism [7]. From a pathological point of view, however, the first phases of the development of D2T are linked to gradual and progressive insulin resistance, and occur in parallel with the hyperfunction of the pancreatic beta cells, which, in an attempt to compensate for the reduction in the effect of insulin on peripheral tissues, increases their production. Subsequently, however, with the loss of the insulin reserve, the individual reaches the stage of full-blown diabetes and, therefore, a greater risk of organ damage [8,9]. Diabetes, however, should not be considered only a metabolic condition with a relative loss of insulin function; it largely owes its genesis to a low-grade local chronic inflammatory state (meta-inflammation), which is linked to the production and release of multiple inflammatory cytokines, such as interleukins and tumor necrosis factor [10]. Many inflammatory markers have been related to obesity and a large study demonstrated the existence of a link between body composition and systemic inflammatory markers [11]. Other studies have supported similar claims regarding the erythrocyte sedimentation rate [12], plasminogen-activator inhibitor 1 [13], and some inflammatory cytokines [14,15], reinforcing the role of the interaction between inflammation and glucose metabolism. It remains to be established how meta-inflammation influences glucose metabolism and, recently, the answer was complicated by the knowledge obtained from the study of the functions of the human intestinal microbiota. The intestinal microbiota has a symbiotic relationship with the host by acting as a driver of inflammation by mediating the absorption of certain nutrients, which then contributes to metabolic pathologies [16,17]. Evidence of this role of the intestinal microbiota is contained in several studies in which obesity and T2D are associated with alterations in the intestinal microbiota [18,19,20]. At the intestinal level, the microbiota produces a series of metabolites, such as short-chain fatty acids (SCFA), increases the biosynthesis of vitamins and amino acids, and participates in the turnover of bile acids, as well as cell–cell interaction with the other components of the host [21]. Therefore, the balance in the cellular composition of the intestinal microbiota plays an important role in the host’s metabolism and the development of insulin resistance and T2D obesity (Figure 1); on the other hand, the relationships with the main cells of the immune system also change, at the level of the intestinal wall. In this review, we explain the dense web of connections between the gut microbiota and inflammation, insulin resistance, and glucose-metabolism diseases.

## 2. Visceral Adipose Tissue—Beta-Cell Interaction

Visceral adipose tissue has long been recognized as having a key role in the onset and maintenance of insulin resistance, beta-cell dysfunction, and increases in cardiovascular risk [22], because the adipocytes organized together in the fat deposits of the human body are considered metabolically active cells, capable of influencing the activity of beta cells in the production of insulin, causing the release of adipokines in connection with the presence of an inflammatory state, resulting in lipotoxicity.

Adipose tissue represents a real endocrine gland, and it is an important source of bioactive hormones, which are key factors in beta-cell function and impairment.

Leptin exerts direct effects on pancreatic beta cells, stimulating the Janus-kinase (JAK)/signal transducer of activation (STAT)—the mitogen-activated protein kinase (MAPK) signaling pathway [23]. This adipokine prevents apoptosis and beta-cell dysfunction [24,25].

In addition, leptin can alter beta-cell function and induce apoptosis by stimulating the release of interleukin-1b (IL-1b), inhibiting the expression of the IL-1-receptor antagonist [26], and activating c-Jun N-terminal kinase (JNK) [27]. Other adipokines have also been related to protective and anti-apoptotic effects on beta cells. For example, low levels of adiponectin have been associated with insulin resistance and beta-cell dysfunction [28]. Furthermore, adipsin [29], visfatin [30], irisin [31], omentin [32], and apelin [33] have been shown to have a protective effect on beta cells. On the other hand, some adipokines have a negative impact on pancreatic beta cells. Thus, resistin both induces insulin resistance and impairs insulin secretion in pancreatic beta cells [34], as well as tumor necrosis factor α (TNF-α) [35] and fetuin-A [36]. The novel adipokines asprosin and retinol-binding protein 4 (RBP4) were reported as important features of the pathophysiology of T2D and beta-cell dysfunction in preclinical studies and animal models [37,38].

In addition, visceral adipose tissue is able to release free fatty acids (FFA) into the circulation through the mechanism of lipolysis. These FFAs are important sources of energy during fasting [39]. However, chronically elevated levels of FFAs inhibit glucose-stimulated insulin secretion and lead to beta-cell dysfunction by activating specific signaling pathways involved in glucose metabolism, insulin resistance, and beta-cell function [40] through cytotoxic mechanisms, causing beta-cell apoptosis [41,42].

As mentioned above, diabetes is characterized by a state of chronic low-grade metabolic inflammation (local and systemic), also called meta-inflammation, which has been shown to contribute to the development of insulin resistance and progression to T2D and is characterized by the abnormal expression and production of multiple inflammatory cytokines, such as interleukins [10].

Visceral adipose tissue, through the production of several cytokines and proinflammatory factors such as IL-2, IL-6, IL-8, IL-12A, or monocyte chemoattractant protein-1 (MCP-1), can play a key role in the alteration of beta-cell function [43,44].

Specifically, peripancreatic adipose tissue, due to its close proximity to the islets of Langerhans, is implicated in beta-cell dysfunction through paracrine mechanisms. The most important mediators of this interaction include several factors, such as the chemokine (C-X-Cmotif) ligands (CXCLs)-1, -2, -3, and the induction of the CXCL-5/lipopolysaccharide CXC chemokine by (LIX), which act on the CXC receptor 2 [45].

Activated macrophages infiltrating adipose tissue [46,47] and inflamed adipocytes can also lead to harmful effects and induce beta-cell death [48].

Finally, B2 lymphocytes, adipose-resident immune cells, adipocyte mitochondrial dysfunction, and reactive oxygen species (ROS) can increase insulin resistance [49] and contribute to beta-cell impairment [50].

## 3. Gut Microbiome and Metabolism

### 3.1. Inflammation and Insulin Resistance

The human gastrointestinal tract contains a complex community of trillions of microbe, collectively known as the gut microbiome. These microbes carry out important physiological functions, such as nutrient metabolism, energy harvest, the regulation of immunity, and the maintenance of mucosal defense. Mounting evidence suggests a causal link between altered gut microbiome composition, known as gut dysbiosis, and the development of human diseases such as adipose-tissue dysfunction and insulin resistance/T2D [20,51]. A mild inflammatory state is usually sustained by an increase in circulating pro-inflammatory cytokines in patients with metabolic syndrome, eliciting metabolic effects such as insulin resistance and glucose intolerance; this occurs more frequently in the course of bacterial infections or chronic inflammation of the upper airways [52,53,54]. Results derived from animal and human models have demonstrated that experimental pharmacological treatments obtained from the activity of E. Coli, with anti-inflammatory effects, had positive effects on glucose tolerance [55,56,57,58]. Other anti-inflammatory cytokines, such as IL-4 can increase glucose tolerance and inhibit adipogenesis and macrophage activation [59,60]. The cytokine IL-13 encourages macrophage-alternative activation [61]. Experimentally, it has been shown that both cytokines are elevated in obese and sedentary people, as well as in those with high insulin resistance [62,63]. This anti-inflammatory activity appears to be thwarted by the refractoriness of the receptor of these interleukins, which is frequently found in patients with metabolic syndrome [51]. In adipose tissue, there are two major populations of the macrophages M1, classically activated macrophages, and M2, alternatively activated macrophages. In obese patients, M1-macrophage numbers increase and are correlated with adipose-tissue inflammation and insulin resistance. In contrast, M2 macrophages exert anti-inflammatory effects and utilize oxidative metabolism to maintain adipose-tissue homeostasis. The M1 macrophages are generally responsible for the secretion of pro-inflammatory cytokines and are associated with the development of type II diabetes by altering local and distant tissue functions. Some cytokines can attract immune cells to metabolically active tissues, such as monocyte chemoattractant protein-1 (MCP1), which is increased in the adipose tissues of obese individuals and induces insulin resistance [64,65,66,67].

Therefore, insulin resistance is the determinant event in a chronic inflammatory state that mainly involves the participation of macrophages, which, at the same time, contribute to the development of diabetes [68]. Confirming the role of macrophages in diabetes mellitus, it was observed that the gradual decrease in the functional reserve of beta cells at the level of the pancreatic islets is driven precisely by an inflammatory infiltrate with a prevalent monocyte–macrophage component [69,70,71]. On the other hand, it was shown that by attenuating the intensity of the inflammatory response, with the use of an IL-1 receptor antagonist, there was an improvement in the inflammatory infiltrate affecting the pancreatic islets, improved preservation of beta-cell junctions and, therefore, less insulin resistance [56]. Macrophages also play a crucial role in causing inflammation in the liver during obesity [72]. The pro-inflammatory role of macrophages has not only been demonstrated in the pancreatic islets, but also in other organs involved in glucose metabolism, such as the liver. In fact, the presence of an inflammatory infiltrate specifically linked to the pro-inflammatory activity of the macrophages encourages damage to hepatocytes, causing insulin resistance, hepatic steatosis, and type 2 diabetes [73]. In this process, the role played by macrophages in hepatocytes is not only inflammatory, but also metabolic, as they are also capable of producing insulin-like growth-factor-binding protein 7 (IGFBP7), which competes directly with the insulin receptor, compromising the signal transduction coupled to this receptor [74]. All this suggests that the immune system is involved in the proper functioning of glucose metabolism and, therefore, the pathological conditions related to it. This demonstrates that the chronic low-grade inflammation observed in obesity and type 2 diabetes has harmful consequences for human metabolism and acute inflammatory responses to pathogens further worsen insulin resistance and glycemic control.

### 3.2. Gut Permeability and Insulin Resistance

Inflammation is a biological response of the immune system, which can be triggered by exposure to pathogens. In particular, bacterial components, such as lipopolysaccharides (LPSs), are sources of metabolic inflammation, as LPS has been found to be increased in the circulation of people with diabetes [75]. The LPSs use increased intestinal permeability to enter the circulation [76]. This not only results in a systemic inflammatory response, but also influences glucose-tolerance mechanisms by inducing hepatic insulin resistance and impairing glucose-stimulated insulin secretion [77]. Two phenomena have been observed in the course of acute inflammation: “metabolic endotoxemia” and “postprandial inflammation” [78,79]. The former is an inflammatory response to increased systemic LPS levels due to a “leaky gut” [77]. Postprandial inflammation is instead the increase in circulating endotoxins and other inflammatory markers after meals, especially high-fat meals [80,81,82,83,84].

### 3.3. Intestinal Microbiota in Metabolic Diseases

The intestinal microbiota plays a key role in digestion, in the production of metabolites potentially capable of altering human metabolism, and in the development of the immune system; the latter in particular is involved in the genesis, of obesity and type 2 diabetes, as well as predisposing individuals to these conditions [85]. 

An interesting explanation for the involvement of the intestinal microbiota in the genesis of T2D is given in a recent review [86], which indicates how the bacterial composition plays an important role in the genesis of metabolic diseases through sedentary lifestyles and high-fat diets. The key point is that the development of T2D is a multistep process starting from obesity. In this phase, a diet rich in certain foods, such as fats, tends to shift the balance of the microbiome towards dysbiosis and, therefore, to an increase in insulin resistance and inflammation, which, as stated above, are the two factors determining T2D. 

The action elicited by various bacterial species belonging to the intestinal saprophytic flora is to continuously stimulate the biological reactivity of the intestine-associated lymphoid tissues (GALT), stimulating the production of immunoglobulins and their macrophagic activity [87]. This dynamic relationship with the immune system results in an increase in microbial metabolites, such as short-chain fatty acids (SCFAs) or components such as DNA and polysaccharide A (PSA) [51]. In general, there is currently no definition of a “healthy gut microbiota.” Studies deriving from mouse models have shown that mice deprived of intestinal germs showed themselves to be free from obesity despite being subjected to a caloric diet, while, in contrast to this, the introduction of bacterial species related to obesity such as those of the Bacteroides class led to weight gain, and reduced glucose tolerance increased insulin resistance, leading to the accumulation of adipose tissues and a greater acceleration of the atherosclerotic process [88,89,90]. These studies suggest that the transfer of a microbiota related to an obese phenotype in a bacteria-free subject leads to the acquisition of an obese phenotype; therefore there is a causal relationship between the intestinal microbiota and metabolism. Since there is no definition of a healthy microbiota, it is possible to associate some pathologies, such as glucose metabolism disorders, with increases in less beneficial species, such as Bacteroides, or with a loss of diversity among the various species with the expansion of microorganisms that are usually underrepresented (often opportunistic pathogens) [18,91,92]. Diet, lifestyle, and antibiotic use have been identified as triggering events for these changes [93,94]. In the case of type 2 diabetes, an increase in the pro-inflammatory bacterial tiller type was found at the expense of anti-inflammatory bacteria in T2D [18,91,92]. Thus, an increase in all these pro-inflammatory Gram-negative bacterial species could be a plausible source of the meta-inflammation observed in metabolic diseases [95]. The beneficial effects of a saprophytic and balanced bacterial flora are essentially linked to the production of SCFAs [18]. These are derived from the microbial degradation of fibers, and they exert several beneficial effects on host metabolism. In diabetes, SCFA production is reduced [18,96]. A recent study found a causal relationship between a genetic increase in butyrate production and improved insulin response [97].

## 4. Gut Barriers and Metabolic Disorders

The synergism between the intestinal microbiota and the immune system associated with the intestinal mucosa constitutes the intestinal barrier. It is a fundamental component in the health of the individual, as its correct functioning prevents the infiltration of bacteria and their translocation from the intestine to the systemic circulation and, therefore, it affects the chronic inflammatory state, which is considered essential in glucose metabolism [98]. The function of the intestinal barrier is essential to prevent insulin resistance a correlation has been firmly established in animal models between the latter and the levels of circulating LPS, considered reliable markers of bacterial translocation [77]. Further proof of the link between bacterial translocation and insulin resistance is the fact that a treatment based on antibiotics simultaneously reduced the levels of intestinal and systemic LPS and improved glucose tolerance [99]. Increased intestinal permeability [100] is a phenomenon intimately related to the tight junctions that anchor the cellular elements of the intestinal wall. They are made up of protein complexes that prevent the leakage of various compounds along the paracellular spaces [100]. In addition, LPS has been shown to directly increase intestinal permeability in vitro and in vivo in mice, suggesting a link between increased intestinal LPS and the dysfunction of these junctions [101]. The loss of intestinal-barrier integrity automatically impairs glucose absorption in the intestine. This occurs through the Glut-2-dependent reprogramming of intestinal epithelial cells [102]. Although these phenomena are very evident in animal models, human studies showing the link between increased intestinal permeability and a disturbance of glucose absorption linked to the dysfunction of the tight junction are still lacking. Glut-2-dependent intestinal epithelial cells are active glucose transporters that owe their proper function to the integrity of the adherent tight junction. Consequently, the disruption of the barrier leads to Glut-2 dysfunction, hyperglycemia and, thus, to a systemic influx of microbial products and the increased spread of enteric germs [102].

### 4.1. The Interaction between Intestinal Immune System and Microbiota: The Role of Immunoglobulins

The GALT system can produce numerous immunoglobulins capable of eliciting an immune response. The induction of the production of such molecules is essential to build immune tolerance against intestinal bacteria and to prevent the effect of the metabolism of these bacteria through the production of toxins [103,104]. Immunoglobulins control the intestinal microbiota and prevent bacterial invasion by binding directly to microorganisms to block their contact with the host organism. Furthermore, the immunoglobulins bind to the bacteria to carry out “opsonization,” or to facilitate their phagocytosis by the dendritic cells that are formed in their function, by presenting the antigen. The class of immunoglobulins that is most active in controlling the microbiota is secretory IgA [105]. In particular, a deficiency of secretory IgA has been observed in obese mice and is associated with reduced glucose tolerance, the greater presence of activated macrophages in adipose tissue, and a higher level of systemic endotoxins [106]. In the same study [106], it was observed that by subjecting the mice to an antibiotic therapy, an improvement in glucose tolerance was obtained, and by using metformin or bariatric surgery, a better response of the intestinal microbiota to secretory IgA was ensures. The T cells, especially helper T cells, are the most important building blocks of proper immune and antibody responses [107,108]. The exhaustion of their function can lead to the development of metabolic syndrome. Immunoglobulins are produced by B cells, which had impaired function in T2D [109]. Furthermore, B cells also accumulate in the visceral adipose tissues of obese and diabetic mice and are capable of producing more pro-inflammatory cytokines than B cells in the adipose tissues of lean controls [110]. Bacterial translocation explains the role of these cells in the development of inflammation in obese and diabetic patients, leading to insulin resistance. However, this concept is currently only confirmed in the animal world, while there is a lack of clear evidence in humans [51].

### 4.2. Meta-Inflammation: Molecular Mechanism and Microbiota Activity

In humans, the connection between the microbiota and meta-inflammation in metabolic diseases has only recently been established. 

**Figure 2:**
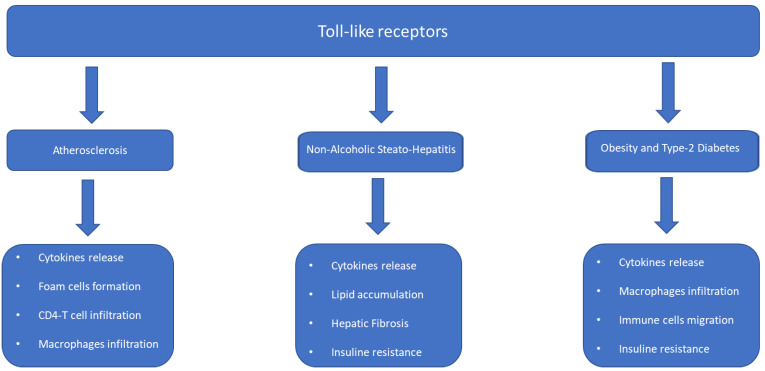
Toll-like receptors and metabolism.

Toll-like receptors (TLRs) are a class of proteins with a front-line receptor function in the recognition of the antigen belonging to microorganisms from the outside, but they are also capable of activating themselves in response to antigens released by cells in apoptosis or in damaged tissues. For this reason, they always play a bridging role between innate and adaptive immunity. In particular, TLR2 and TLR4 are the subgroups of Toll-like receptors (TLRs) that have been identified as responsible for the development of metabolic syndrome and type 2 diabetes mellitus (Figure 2). Insulin infusion is capable of suppressing TLR4 [105], suggesting that, in addition, these molecules are associated with insulin resistance; high concentrations of insulin are also present in the pancreatic islets of obese animal models [111,112,113]. The TLR5 subgroup has also been associated with the development of metabolic diseases, particularly the flagella, which are involved in bacterial motility [114]. In addition, TLR5 is also probably involved in the development of meta-inflammation, since it is associated with both low-grade inflammation and metabolic syndrome, which are reversible after antibiotic therapy [115]. Similar to the TLR system, the nucleotide-binding oligomerization domain contains the proteins NOD1 and NOD2, which are cytosolic receptors that respond to bacterial peptidoglycans and have been associated with the development of insulin resistance [116,117]. Direct NOD1 activation led to insulin resistance while, conversely, NOD2 signaling is protective against Type 2 diabetes. The poor functioning of NOD2 increases inflammation and insulin resistance [118]. The activation of NOD2 via bacterial cell-wall-derived muramyl dipeptide (MDP) improved insulin resistance [119]. It appears that NOD2 signaling is beneficial for, while NOD1 has deleterious effect on insulin sensitivity and beta-cell function.

Inflammasomes are made up of a few protein subunits present in the cytoplasm of all cells that play a role in innate immunity in the human body and are involved in the inflammatory response [120,121]. It is precisely from these protein oligomers that the production and release of true types of interleukin are derived, through proteolysis. In particular, in the development of the metabolic syndrome, interleukins 1β (IL-1β) and 18 (IL-18) are the most frequently considered factor in the union between glucose metabolism and the immunological function of the intestinal microbiota [122]. This is further demonstrated by the fact that the activation of proteins belonging to the inflammasome is generated by the activity of receptors in the recognition of molecular residues associated with pathogens of microbial origin (PAMP) or molecular patterns associated with hazards (DAMP) released by the host cell [123]. The pattern-recognition receptors involved in inflammasomes include NLR (nucleotide-binding oligomerization domain and leucine-rich repeat-containing receptors). In the context of the inflammasome NOD-like receptor family, pyrin domain 3 (NLRP3) is the most active in responding to pathogenic stimulation [124]. Several studies have linked increased NLRP3 expression in adipose tissues and monocytes to obesity and T2D [125,126]. A decrease in NLRP3 activation in adipose tissue coincided with reduced inflammation and improved insulin sensitivity [127]. Therefore, NLRP3 is considered an important component, forming a link between inflammation and metabolic disease. More recently, NLRP12 was implicated in the development of metabolic diseases due to its effects on intestinal inflammation and promotion of the growth of beneficial bacteria [128,129]. The protective effect of NLRP12 expression does not end in the intestine, but continues in the adipose tissue, where its high concentration is negatively associated with obesity, while, by contrast, its deficient function induces adipose tissue deposition, insulin resistance, and the need for inflammatory macrophages [130]. Furthermore, other inflammasomes are involved in meta-inflammation, but their exact mechanism of action is still unknown. Finally, some anti-inflammatory cytokines have ben found to be essential elements in the maintenance of intestinal homeostasis. Although several cytokines have been associated with meta-inflammation in obesity and T2D, only a few, such as IL-22, IL-23, and IL-36, were shown to have a direct connection to the gut microbiota [131].

### 4.3. Microbiota Metabolites and Inflammation and Insulin Resistance

The intestinal microbiota can also participate in glucose metabolism through the production of metabolites, such as imidazole propionate, which can be absorbed by the intestine and elicit biological actions in this sense [132,133]. In this context, however, only a limited number of them have an established relationship between microbiota and glucose metabolism, while for some others, the data are still insufficient. Short-chain fatty acids (SCFAs), as already argued, are direct products of intestinal-microbiota metabolism. Their abundance is linked to greater glucose tolerance and the prevention of T2D [134]. The presence of an adequate pool of SCFAs also has beneficial effects on glucose metabolism as, since they are direct metabolites of enterocytes, they improve the integrity; furthermore, by encouraging the development of polymicrobial flora, they also increase the efficiency of the intestinal barrier [135]. Butyrate increases intestinal epithelial integrity [135], the development an anti-inflammatory environment, resistance to enteropathogens, and the generation of regulatory T cells. Furthermore, butyrate inhibits the epigenetic modulator histone deacetylase (HDAC), inducing a sustained anti-inflammatory response in intestinal cells and other tissues [136,137]. This has been shown to translate into a decrease in chronic inflammation and, therefore, better glucose tolerance. Furthermore, SCFAs have been implicated in the regulation of appetite [138] and the improvement of resistance in peripheral tissues [139]. It must be noted that although most of these molecules have beneficial effects on glucose metabolism, others instead show opposite effects, as evidenced in animal model studies [140]. Bile acids are metabolized by the intestinal microbiota and are involved in glucose metabolism [141]. They are efficiently absorbed into the enterohepatic circulation. Small parts leave the circulation and are excreted in the feces or appear in the systemic circulation, with receptors expressed in hepatocytes [141]. The experimental use of hepatocyte-bile-acid-receptor agonists induced GLP-1 secretion and improved glucose tolerance in mice [142]. An antibiotic treatment reversed this metabolic phenotype, suggesting the involvement of the gut microbiota [142]. Again, there is a need to confirm these data in humans. Trimethylamine-N-oxide (TMAO) is produced by the liver from trimethylamine (TMA), which is in turn produced by the intestinal microbiota from nutrients containing choline and carnitine. The TMAO has been considered responsible for the chronic inflammatory state underlying the development of atherosclerotic plaque. A recent human study found that low-calorie diets depleted choline and L-carnitine. These changes were associated with improvements in baseline insulin values and insulin resistance in overweight and obese adults [143]. All three metabolites (TMAO, choline, and L-carnitine) have been associated with diabetes and gut-microbiota activity [144,145].

Obesity is not the only condition predisposing individuals to T2D, but some pieces of evidence also indicates the importance of the role of body composition in the appearance of metabolic disturbances. It is important to balance the intake of certain amino acids with a diet focused especially on the quantity and quality of protein intake. The improvement of strength and the efficiency of muscular work leads to an increase in metabolically active body mass, which may have important implications in the development of T2D [146].

The integration, above all of hydroxyl-methyl butyrate (HMB), L-leucine (Leu), L-glutamine (Gln), and L-arginine (Arg) seems to have important repercussions in improving insulin resistance. On the other hand, the sarcopenia also appears to be related to the development of T2D [146].

## 5. Changes in the Gut Microbiota in Metabolic Diseases

To summarize, the main changes identified in the gut microbiota in the course of metabolic diseases are as follows:

- Decreases in the number and quality of microbial subpopulations. Decreases in this diversity lead to an imbalance known as dysbiosis.
- Changes in the functional capacity of the microbial flora, resulting in changes in the production and metabolism of various compounds, such as short-chain fatty acids (SCFA), bile acids, and trimethylamine-N-oxide (TMAO), which can affect host metabolism and inflammation.
- Dysbiosis linked to an increase in Bacteroides species induces an increase in the production of lipopolysaccharides (LPS). Increased LPS levels in the intestine induce meta-inflammation and insulin resistance, encouraging the development of metabolic diseases.
- Dysbiosis induces increased intestinal permeability, contributing to barrier dysfunction and allowing the translocation of bacterial components, such as LPS, into the bloodstream, triggering systemic inflammation and metabolic disturbances.
- Alterations in gut-microbiota composition can influence dietary calorie extraction and affect fat storage and distribution, contributing to obesity and metabolic dysregulation. Indeed, the increase in the Firmicutes-to-Bacteroidetes ratio is associated with a greater extraction of energy from the diet and a greater deposition of fat mass.

## 6. Limitations

Despite the increasing number of tests demonstrating that the intestinal microbiota is the driver of inflammatory processes and metabolic diseases, there are still many problems of a practical nature.

This problem is due both to the fact that most part of the knowledge is based on the extraction and manipulation of fecal DNA, which makes an accurate study of all microbial species difficult, and to the fact that an ideal microbiota with which to maintain the intestines has not yet been established.

Another limitation of current knowledge is that the intestinal microbiota is a dynamic entity, which undergoes changes as a function of multiple factors; this makes its composition variable not only over time, but also in the different sections of the digestive tract [147,148]. A human study found that stool consistency is a more important factor in variation of the fecal microbiota than medication use, early childhood events, and diet [149].

Another important limitation is the fact that in most of the studies, the experiments were conducted on animals, but these experimental conditions are not similar and, therefore, faithfully reproducible in humans. Compared to animals, humans have a different, more varied diet, influenced using chemical products for conservation. Humans habitually cook their food and often make use of numerous food-preservation techniques whose use would be unthinkable in the animal world [149].

Another limitation could be linked to the fact that the use of inflammatory markers correlated with the intestinal microbiota does not consider the presence of other possible comorbidities, the chronic or acute use of drugs, lifestyle, or diet [150]. The presence of these numerous confounding factors limits the validity of some of the data and reinforces the need to obtain new evidence to better understand the interconnection between the microbiota, inflammation, and glucose metabolism.

## 7. Conclusions and Future Directions

In summary, changes in gut microbial composition and lower microbial diversity in obese subjects were associated with higher levels of inflammatory. This implies the role of the gut microbiota in the low-grade inflammation seen in people with metabolic syndrome. The intestinal microbiota seems to play an important role in glucose tolerance, but more data supporting this finding are need through further testing in order to overcome the practical difficulties involved in the numerous confounding factors that interfere with the bacterial and immune population residing in the intestine. Different pathogenetic mechanisms and molecules have been studied to better understand the effects of the intestinal microbiota on the inflammatory state and on glucose metabolism; however, conclusive evidence from humans has not yet been obtained. Considering that there is no normal composition of the microbiota, subsequent studies with new experimental methods and a greater focus on humans will need to clarify the ideal microbial composition, and whether it even exists, and the role it plays in the chronic inflammatory state and in metabolism.

Subsequent studies should expand the number of biomarkers and increase their sensitivity to improve the understanding of the mechanisms linking the gut microbiota, inflammation, and metabolic diseases. In this sense, it is necessary to identify more specific markers that not influenced by other superimposed processes, such as other infections or other pathological conditions. Once more specific markers have been obtained, it will be possible to proceed with studies to introduce prebiotic and probiotic therapies to improve the composition of the intestinal microbiota. It will also be useful to more closely study the role of body composition and the quality of foods used in human nutrition to obtain an overall view of the link between the host and the intestinal microbiota.

## Data Availability

Literature search.
